# Commentary: Greater Emotional Gain from Giving in Older Adults: Age-Related Positivity Bias in Charitable Giving

**DOI:** 10.3389/fpsyg.2016.01075

**Published:** 2016-07-14

**Authors:** Mary B. Hargis, Daniel M. Oppenheimer

**Affiliations:** ^1^Department of Psychology, University of California, Los AngelesLos Angeles CA, USA; ^2^Department of Psychology and Anderson School of Management, University of California, Los AngelesLos Angeles CA, USA

**Keywords:** charity, aging, older adults, gist based processing, donations

Despite the fact that the population of is rapidly aging (Ortman et al., 2014), until recently scholars of charity have largely ignored older adults. Bjalkebring et al. (2016) begin addressing this gap in the literature. By showing that increasing age is associated with stronger feelings of sympathy and compassion and that older adults are more likely to report feeling positive emotions when making donations, Bjalkebring et al. (2016) connect the literature on age-related positivity biases to the literature on philanthropy. We endorse this approach of identifying ways in which older populations differ from younger populations and exploring how those differences might inform donation behavior. Indeed, this tactic has the potential to reveal a large number of age-related differences in giving beyond those based on an age-related positivity bias.

It makes sense that Bjalkebring et al. (2016) focused primarily on emotional differences between older and younger adults, as the bulk of the literature on charitable giving has shown that people often give to charity for emotional reasons, and respond more strongly to emotional (than rational) appeals for donations (e.g., Loewenstein and Small, 2007; Huber et al., 2011). In fact, there are other emotional differences aside from positivity biases that have implications for donation behavior. For example, older adults often experience greater emotional complexity—that is, experiencing positive and negative emotions together (Carstensen et al., 2011). Therefore, it may be productive to interpret the higher levels of sympathy and compassion older adults reported in Bjalkebring et al. (2016) experiment 1 through the lens of older adults' improved ability to deal with emotional complexity above and beyond any bias toward purely positive emotions.

However, as important as emotion-related differences are to the study of aging and philanthropy, it is worth noting that there are also a number of cognitive factors that influence both older adults' processing of emotional information (such as manipulations of cognitive resources or goals; Reed et al., 2014; Reed and Carstensen, 2015), and giving more generally. Thus, exploring age-related cognitive differences has the potential to reveal additional insight as to how donation behavior changes across the lifespan.

Aging is related to cognitive declines in processing speed (Salthouse, 1996) and working memory capacity (Hasher and Zacks, 1989). Importantly, older adults are often able to compensate for these declines by directing cognitive resources toward information of high value (Castel, 2008). Younger adults sometimes find it difficult to discriminate between high-value information and low-value information (Castel, 2008), suggesting that they may not be as effective as older adults in identifying the worthiest causes or the most impactful ways in which to give. If older adults are sensitive to value when making spending decisions, they might be more likely than younger adults to donate, especially to causes they view as important. Older adults may also be better able to effectively determine the highest-value donation in terms of efficiency, personal value, or societal outcomes (for a discussion of younger adults' errors in optimizing the value of donations, see Baron and Szymanska, 2011).

Another strategy that older adults may use in light of cognitive declines is to engage in more gist-based processing, especially of monetary information (Castel, 2005). Rather than recalling exact details about studied items, older adults tend to remember general representations of those items, such as approximate value (e.g., remembering that an item costs “about $4.00” rather than “exactly $3.92”), or relative value (e.g., which item cost less than another; Flores et al., 2016). Gist-based processing may have implications for how older adults decide when, how much, and to which causes they donate. If older adults make charitable giving decisions using gist, they may derive warm glow simply from the act of giving, irrespective of the exact dollar amount they donated. Younger adults' more accurate memory for exact prices may lead to different emotional responses to donations of different dollar amounts (for a discussion of the function mapping giving to utility in younger adults, see Strahilavitz, 2011). Furthermore, one of the most widespread “take home” messages that practitioners have derived from the scientific literature on philanthropy is that people are more likely to give (and give larger amounts) when presented with specific, tangible information about a charity's outcomes, as compared to more general information (e.g., giving a milking cow to a particular family, as opposed to general “poverty alleviation”; Cryder et al., 2013). If older adults process information using gist, their giving behavior may be less affected by tangibility than younger adults, as specific details may be lost in favor of a gist-based representation. In other words, common strategies for charitable appeals may be less effective on older populations.

Scholars should also pay attention to a number of philanthropy-relevant demographic differences across age groups. For example, older adults' net worth is significantly higher on average than younger adults' (Taylor et al., 2011), and income is a major factor in charitable giving (Gittell and Tebaldi, 2006; Choi and Chou, 2010). Similarly, religious participation has been positively correlated with giving behavior (Jackson et al., 1995) and may be related to age (Davie and Vincent, 1998). These demographic differences are worth considering both because they themselves may be interesting avenues for exploring age-related differences in donation behavior, but also because they represent potential confounds in (inherently quasi-experimental) investigations on how aging affects giving. As noted by Bjalkebring et al. (2016), these sorts of confounds can make it difficult to distinguish between influences of aging, *per se*, and cohort effects.

In sum, findings by Bjalkebring et al. (2016) demonstrate how cognitive aging can influence charitable decision making and serve as motivation for many future avenues of exploration, including how emotional complexity, subjective value, and gist-based processing may affect charitable giving in younger and older adults. Established theories of cognitive aging can be a fruitful source of novel questions about how donation behavior changes across the lifespan, and may have applications for developing charitable appeals aimed at a rapidly increasing segment of the population.

## Author contributions

The ideas and arguments were jointly developed by MH and DO. MH drafted the manuscript. Both authors edited the manuscript extensively.

## Funding

The first author is partially supported by National Institutes of Health (National Institute on Aging), Award Number R01AG044335.

### Conflict of interest statement

The authors declare that the research was conducted in the absence of any commercial or financial relationships that could be construed as a potential conflict of interest.
